# A Thermopile Detector Based on Micro-Bridges for Heat Transfer

**DOI:** 10.3390/mi12121554

**Published:** 2021-12-13

**Authors:** Na Zhou, Xuefeng Ding, Hongbo Li, Yue Ni, Yonglong Pu, Haiyang Mao

**Affiliations:** 1 Institute of Microelectronics of Chinese Academy of Sciences, Beijing 100029, China; zhouna@ime.ac.cn (N.Z.); lihongbo@ime.ac.cn (H.L.); worldly2002@163.com (Y.P.); 2 University of Chinese Academy of Sciences (UCAS), Beijing 100029, China; 3 Jiangsu Hinovaic Technologies Co., Ltd., Wuxi 214135, China; dingxuefeng@hinovaic.com (X.D.); niyue@hinovaic.com (Y.N.)

**Keywords:** thermopile detectors, micro-bridge, poly-Si thermocouples, thermal conduction, CMOS process

## Abstract

A thermopile detector with their thermocouples distributed in micro-bridges is designed and investigated in this work. The thermopile detector consists of 16 pairs of n-poly-Si/p-poly-Si thermocouples, which are fabricated using a low-cost, high-throughput CMOS process. The micro-bridges are realized by forming micro trenches at the front side first and then releasing the silicon substrate at the back side. Compared with a thermopile device using a continuous membrane, the micro-bridge-based one can achieve an improvement of the output voltage by 13.8% due to a higher temperature difference between the hot and cold junctions as there is a decrease in thermal conduction loss in the partially hollowed structure. This technique provides an effective way for developing high-performance thermopile detectors and other thermal devices.

## 1. Introduction

Nowadays, thermopiles, as a type of functional structure, have been widely used in different fields, including some systems and devices, such as non-contact temperature sensors [1,2,3], gas sensors [4,5,6], thermal flow sensors [7,8,9], power generators [10,11,12], and micro-calorimeters [13,14,15]. These thermopile-based devices can be commonly found in military, industry, and consumer electronic domains. Based on the Seebeck effect, thermopile detectors have advantages in being unnecessary of cooling equipment and chopper for operation [16,17]. Therefore, their system design is quite simple when compared with other thermal detectors, such as bolometers and pyrometers. Furthermore, the compatibility with CMOS processes also makes their fabrication process relatively simple and cost-effective. When the novel coronavirus (2019-nCOV) epidemic disease spread all over the world in recent years, thermopiles that can detect infrared radiation in a non-contact way have been demanded globally. 

For decades, thermopiles using continuous membranes (CM) for heat transfer have been commonly investigated, in which the suspended membranes are formed by removing the bulk silicon beneath the film [18,19,20,21,22]. In such a device, heat transfers from the central area to the peripheral areas, namely from the hot conjunctions to the cold conjunctions, along a 360° direction, resulting in low temperature difference between the hot and cold junctions. In order to increase the temperature difference, to improve the performance of the CM-based thermopile detectors, several efforts have been made. In previous reports, black metal materials, black nanomaterials, and metamaterials have been introduced to enhance infrared absorption to improve device performance [23,24,25,26,27]. Nevertheless, the fabrication processes of these materials are complex, and some are incompatible with the CMOS processes. Furthermore, most of the studies have been focused on improving absorption; the methods which directly reduce thermal conduction are rarely investigated. However, the heat conduction of the CM-based detectors is relatively high since the continuous membranes transfer heat easily through dielectric layers, such as SiO_2_ and Si_3_N_4_. Therefore, how to reduce the heat conduction of thermopile detectors by using a CMOS compatible method is still a challenging subject.

To solve the aforementioned problem, this work explores a CMOS-compatible thermopile detector based on micro-bridges (MB) for heat transfer. The micro-bridges are formed to decrease the solid thermal loss to improve the temperature difference between the hot and cold junctions. For comparison, a CM-based thermopile detector is also prepared and tested, and a 13.8% improvement in the performance of the MB device is further confirmed. Compared with other MEMS thermopiles, this device uses materials that are commonly used in CMOS processes, such as poly-Si, Al, silicon oxide, silicon nitride. In addition, the method to form the micro-bridge is simple and can be transferred to any other type of thermal detector. This new method provides a new route to design and fabricate high-performance thermopile detectors.

## 2. Materials and Methods

**Figure 1:**
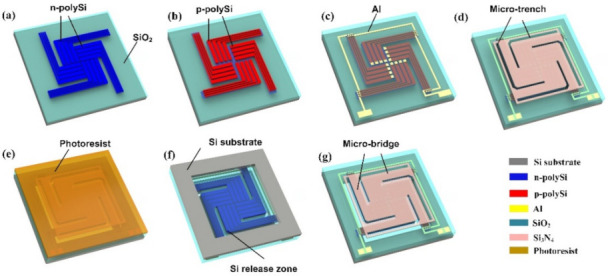
Schematic diagram of the process for fabricating an MB-based thermopile device. (**a**) Deposition polysilicon, implantation (P ion) and pattering, (**b**) Deposition oxide and polysilicon, implantation (B ion) and patterning, (**c**) Metal Al sputtering and patterning, (**d**) Formation of micro-trench, (**e**) Spinning photoresist for protection, (**f**) Deep silicon releasing at the back-side, (**g**) Removing photoresist at the front-side.

The fabrication process for the MB-based thermopile detector is schematically illustrated in Figure 1. All experiments were performed on 200 mm single crystal silicon substrates (p-type, (100), 8–12 Ω·cm). Firstly, a SiO_2_ layer with a thickness of 500 nm and a Si_3_N_4_ layer with a thickness of 300 nm were grown to form the supporting layers on the substrates by PECVD (plasma-enhanced chemical vapor deposition). Then poly-Si layers were deposited, patterned, and implanted with phosphorus and boron ions to form the n-type and p-type thermocouple strips, as shown in Figure 1a,b. The detailed information for the deposition, implantation, and thermal annealing procedures can be found in our previous report [28]. After that, a SiO_2_ layer was deposited and etched, which was further followed by Al sputtering and patterning for interconnection, as exhibited in Figure 1c. Later on, another Si_3_N_4_ layer was deposited for infrared light absorption. Subsequently, part of the dielectric membrane was removed to reveal the silicon substrate by reactive ion etching (RIE), and then photoresist was filled in the trenches on the front side to protect the structure, as can be seen in Figure 1d,e. Finally, the thermopile structure was released by deep reactive ion etching (DRIE) from the back side, as is shown in Figure 1f. Consequently, MB-based thermopile detectors were generated (Figure 1g). Similarly, CM-based thermopile detectors were also prepared with the same back-side-releasing step while the front-side was not previously etched with trenches.

The fabricated structures were characterized by using scanning electron microscopy (SEM) (Hitachi, S-5500). The current-voltage (I-V) curves were measured by using a semiconductor parameter analyzer (Keithley, 4155B) under the radiation of an IR blackbody (HGH, DCN1000H4LT).

## 3. Results and Discussions

### 3.1. Structural Design and Simulation

**Figure 2:**
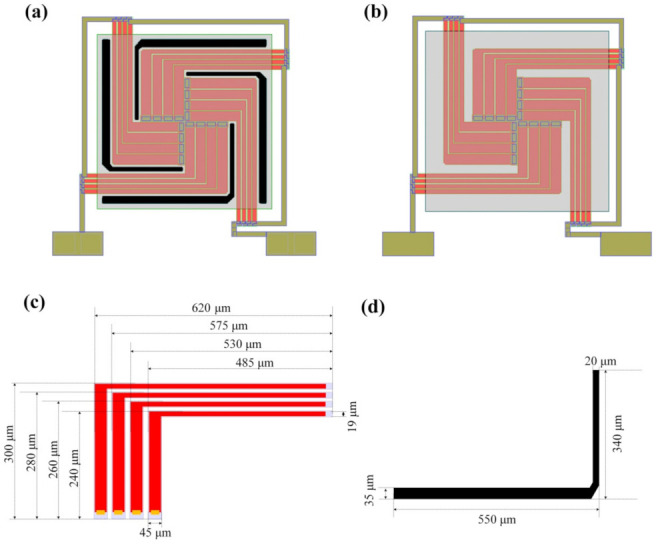
Schematic diagrams of (**a**) an MB-based thermopile, (**b**) a CM-based thermopile, (**c**) a group of thermocouples, and (**d**) a micro trench.

Figure 2a,b shows the schematic diagrams of the MB and CM-based thermopiles. The geometric parameters are shown in Table 1. Both thermopiles consist of 16 pairs of n-poly-Si/p-poly-Si thermocouples in series. The thermocouples patterned on the dielectric membrane are arranged in a pistol array symmetrically for maximizing the utilization of the chip area. The absorption areas of the MB and CM-based structure are 0.5708 and 0.6724 mm^2^, respectively. That is to say, the absorption area of the MB-based structure is about 17.8% smaller than that of the CM structure, as part of the MB absorption membranes are removed to form the holes. The corresponding sizes of a thermocouple group and a micro trench are shown in Figure 2c,d.

**Figure 3:**
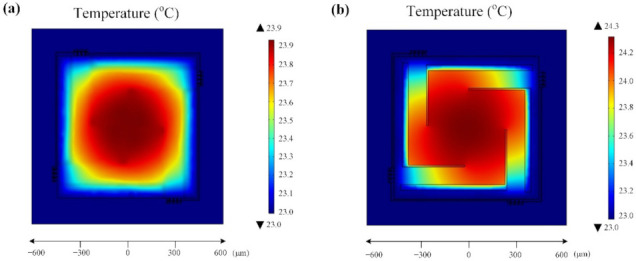
Simulated temperature distributions on the thermopiles. (**a**) The CM-based device, and (**b**) the MB-based device.

By using the finite element method (FEM) software, the 3D models of the CM and MB-based thermopiles were transferred for thermal simulation. In the simulations, the temperature of the cold junctions, also known as the ambient temperature, was set at 23 °C, and the IR radiation flux applied on the thermopiles was set at 100 °C. The ∆T of the different designs are compared. The simulated distribution of steady-state temperature on the two thermopile detectors are shown in Figure 3. As is illustrated, the ∆T in the CM-based thermopile is 0.9 °C (Figure 3a), while that in the MB-based device is 1.3 °C (Figure 3b). This is because in the MB structure, the through-holes contribute partly to cutting off the heat transfer path from the hot junctions to the cold junctions. As a result, the temperature is higher at its hot junctions. 

### 3.2. Profile of the Fabricated Detectors

**Figure 4:**
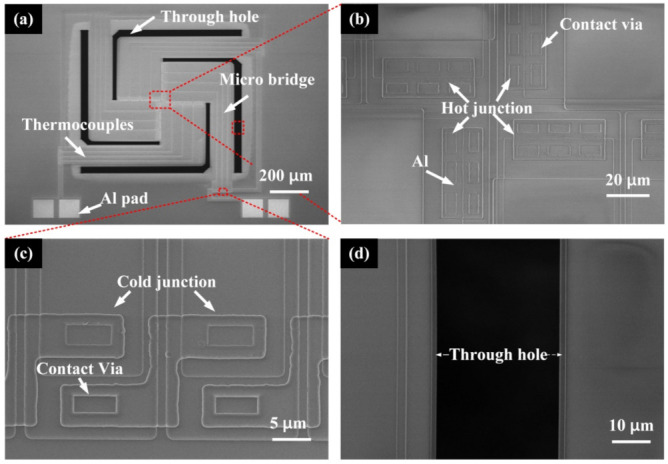
SEM images of (**a**) an MB-based thermopile, (**b**) hot junctions of the device, (**c**) a group of cold junctions in the device, and (**d**) an amplified image of a through-hole.

SEM images of the fabricated MB-based thermopiles are shown in Figure 4a. The through holes could be clearly found around the suspended micro-bridge structures. The SEM image shows that the membranes are flat, which means the micro-bridges have perfect mechanical stability. Figure 4b,c presents the hot and cold junctions of the thermopile detector, where the Al lines are patterned over contact vias. Additionally, a through-hole next to the micro-bridge is amplified and can be seen totally black since it is etched completely, as shown in Figure 4d.

### 3.3. Performance Analysis

**Figure 5:**
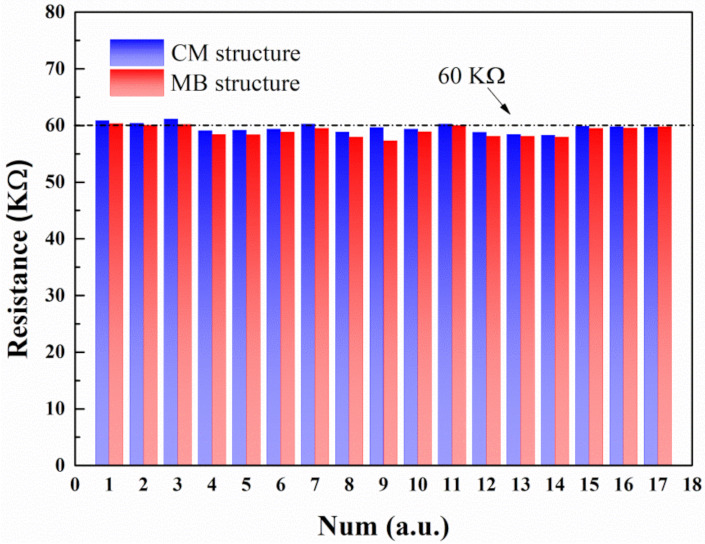
Measured resistances of 34 thermopile devices.

The sheet resistances for the n-type and p-type poly-Si layers were controlled at about 40 and 60 Ω/sq respectively. Figure 5 shows the resistance of 34 different thermopile devices based on the CM and MB structures, respectively. The designed resistance of the thermopile device is 60 KΩ. As is shown in Figure 5, the mean value of the measured resistance is 59.6 and 59.0 KΩ for the CM and MB-based thermopiles, respectively, which are quite close to the theoretical values because of the high uniformity of the CMOS process.

**Figure 6:**
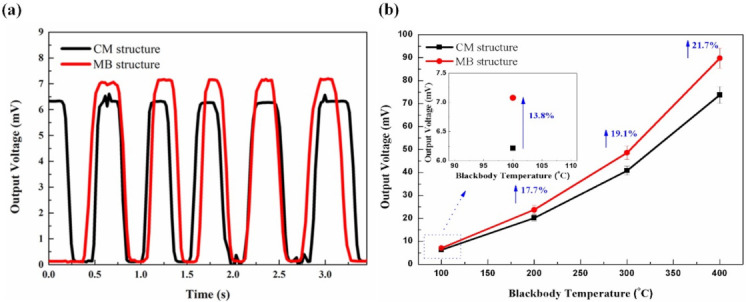
(**a**) Measured response for both types of the thermopiles, (**b**) voltage–temperature response curves of the two thermopiles in a temperature range from 100 to 400 °C. The inset shows the amplified output voltage at 100 °C.

With a 4 Hz chopping frequency and a 7 cm blackbody-detector distance, the voltage responses of the two thermopiles are presented in Figure 6a. The IR response voltage amplitudes for the CM and MB-based thermopiles are 6.22 and 7.08 mV at a blackbody temperature of 100 °C, respectively. Hence, the voltage generated by the MB-based thermopile is 13.8% higher than that of the CM-based thermopile. It should be noticed that the absorption area of the MB-based structure is 17.8% smaller than the CM-based structure. Although the MB-based thermopile detector has less infrared light absorption, it still has a larger output performance due to the decreased heat conduction compared with the CM-based thermopile.

The voltage–temperature response curves of the thermopiles were recorded by changing temperatures of the blackbody from 100 to 400 °C. The results are illustrated in Figure 6b. As can be observed in this figure, the curve of the MB-based thermopile has a much larger growth trend than that of the CM-based thermopile, indicating higher sensitivity of the MB-based thermopile. Moreover, a relative increment of 13.8%, 17.7%, 19.1%, and 21.7% in voltage are achieved for the temperatures of 100, 200, 300, and 400 °C, respectively. It seems that the output responses of these two structures are more distinguishable at higher temperatures. Namely, the performance of the MB-based detectors could be improved more at higher temperatures.

**Figure 7:**
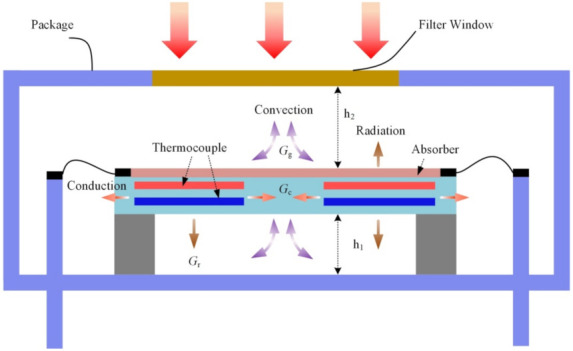
Heat transfer (solid thermal conductance of the structure *G*_c_, convection *G*_g_, and radiation *G*_r_) in a traditional CM-based thermopile.

According to the Seebeck effect, a voltage generated by the device can be expressed as

(1) $$V_{\mathrm{out}}=N\left(\alpha_{A}-\alpha_{B}\right)∆T$$

where *α*_A_ and *α*_B_ are the Seebeck coefficients for the two types of thermoelectric materials. The temperature difference, ∆T, between the hot and the cold junctions is given by

(2) $$∆T=\frac{\eta \varphi_{0}A}{G_{\mathrm{th}}}$$

where *η* is the absorption coefficient, *A* is the absorption area of the detector, *φ*_0_ is the net radiation flux, and *G*_th_ is the thermopile’s total thermal conduction. As shown in Figure 7, the total thermal loss between the hot junctions and their surroundings in a MEMS thermopile detector can be classified into three types, namely the solid thermal conduction, *G*_c_, the gas convection, *G*_g_, and the thermal radiation, *G*_r_, in other words, *G*_th_ can be determined by [29]

(3) $$G_{\mathrm{th}}=G_{r}+G_{g}+G_{c}$$

Thermal radiation refers to the energy radiated from hot surfaces in the forms of electromagnetic waves, and it can be derived by

(4) $$G_{r}=4\varepsilon \sigma AT^{3}$$

where ε is the effective emissivity coefficient of the thermopile structure, σ is the Stefan-Boltzmann constant, and *T* is the environment temperature.

The gas convection loss is due to the heat transport caused by the movement of molecules in air fluids. Thus, $G_{g}$ can be determined by

(5) $$G_{g}=\lambda_{g}\left(\frac{1}{h_{1}}+\frac{1}{h_{2}}\right)A$$

where $\lambda_{g}$ is the thermal conductivity of the atmospheric gas, $h_{1}$ is the distance between the membrane and the cavity bottom, and $h_{2}$ is the distance between the membrane and the box cover of the package.

Solid thermal conduction refers to the dissipation of heat through the suspended micro-structures over the substrate. Since the thermopile structure consists of semiconductor thermocouples and several corresponding layers of dielectric materials,$\;G_{c}$ can be derived by

(6) $$G_{c}=N\frac{\lambda_{1}d_{1}w_{1}}{l_{1}}+N\frac{\lambda_{2}d_{2}w_{2}}{l_{2}}+\frac{\lambda_{3}d_{3}w_{3}}{l_{3}}$$

where *λ*_n_, *d*_n_, *w*_n_, and *l*_n_ denote the material thermal conductivity, the layer thickness, the total width of the layer, and the length of layer (n = 1 refers to n-type semiconductor thermocouple leg; n = 2 refers to p-type thermocouple; n = 3 is for the dielectric layer), respectively. MEMS thermopile IR detectors are generally packaged in a nitrogen environment. Thermal conduction decrement of the MB structure is estimated to come from all three types of thermal loss. Furthermore, the output voltage of the thermopile is proportional to the absorptivity *η* and inversely proportional to the thermal conductance *G*_th_ based on the infrared–electric conversion according to Equations (1) and (2). Therefore, compared with the CM-based thermopile, performance improvement of the MB-based device can be estimated by [30]

(7) $$∆V=\frac{∆T_{\mathrm{MB}}}{∆T_{\mathrm{CM}}}-1=\frac{1+∆A}{1+∆G_{\mathrm{th}}}-1$$

As the absorption area of the MB-based thermopile is 17.8% smaller than the CM-based device, while the output voltage has a 13.8% enhancement, according to Equation (7), it can be calculated that the *G*_th_ of the micro-bridge structure is about 28% smaller than that of the CM structure. Once the temperature is increasing, from Equations (4) and (5), the thermal radiation and gas convection loss of the MB-based structure are lower compared with the CM-based structure since the two parameters are positively changed with the absorption area. Thus, the performance increment is improved with the temperature increasing.

**Figure 8:**
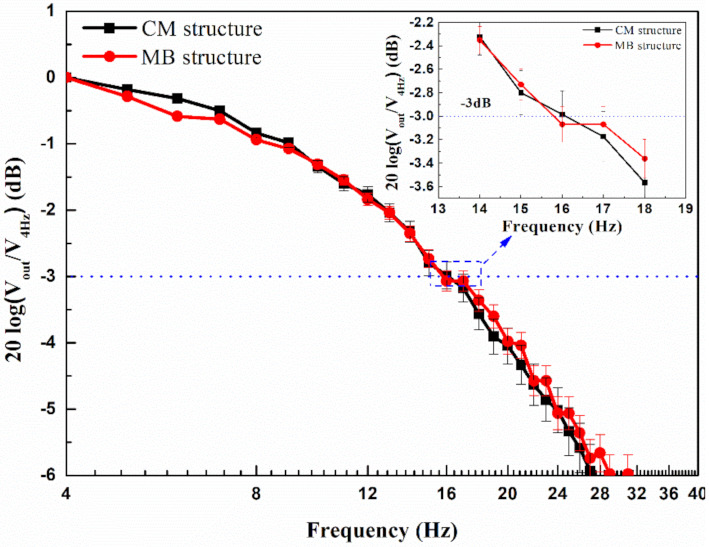
Measured frequency responses of the two thermopiles.

In order to evaluate the response time of the two thermopiles with different structures, the frequency responses were tested, as shown in Figure 8. The chopper frequency was set from 4 to 40 Hz. Based on the tested −3 dB cutoff frequency of 16.0 and 15.8 Hz for the CM and MB-based thermopile, respectively, the response times of 15.6 and 15.8 ms could be obtained based on *t* = (1/4*f*) (Hz). The results exhibit that the MB-based thermopile has a similar response speed to that of the CM-based device.

## 4. Conclusions

In summary, a thermopile detector based on micro-bridges for heat transfer is presented. The detector is fabricated using a standard CMOS process. Compared with a CM-based thermopile detector, the MB structure is able to reduce the thermal conduction of the dielectric membrane. Thus, the thermopile detector exhibits a 13.8% higher performance while maintaining perfect mechanical stability and fast response. These results demonstrate the feasibility of using this new strategy to decrease heat conduction to achieve high-performance thermopiles and other detectors with heat transfer mechanisms.

## Figures and Tables

**Table 1:** Structural parameters of the designed MB-based thermopile.

Structural Parameters	Value
Pairs of thermocouples	16
Device area (mm^2^)	1.44
Absorber area (mm^2^)	0.5708
Thickness of Si_3_N_4_ Absorber (Å)	5000
Thickness of n-type poly-Si (Å)	3000
Thickness of p-type poly-Si (Å)	3000
Trench depth (μm)	2.3
